# Mechanistic classification of isolated severe aortic regurgitation in a contemporary cohort of patients

**DOI:** 10.1093/ehjopen/oeaf042

**Published:** 2025-05-02

**Authors:** Rudy R Unni, Munir Boodhwani, Ibrahim Jelaidan, David T Harnett, Samia Massalha, Calvin Liang, Graeme Prosperi-Porta, David Glineur, Ian G Burwash, Kwan-Leung Chan, Thais Coutinho, Angel Fu, Nadav Willner, David Messika-Zeitoun, Luc Beauchesne

**Affiliations:** Division of Cardiology, University of Ottawa Heart Institute, Room H-3407A, 40 Ruskin Street, Ottawa, ON, Canada K1Y 4W7; Division of Cardiac Surgery, University of Ottawa Heart Institute, 40 Ruskin Street, Ottawa, ON, Canada K1Y 4W7; Adult Cardiology Department, King Faisal Cardiac Center, Ministry of National Guard Health Affairs, P.O. Box 9515, Jeddah 21423, Saudi Arabia; Division of Cardiology, Memorial University, 300 Prince Philip Drive, St. John’s, NL, Canada A1B 3V6; Rambam Health Care Centre, 8 HaAliyah HaShniya Street, Bat Galim, Haifa 3109601, Israel; Department of Medicine, University of Ottawa, 501 Smythe Road, Ottawa, ON, Canada K1H 8L6; Division of Cardiology, University of Ottawa Heart Institute, Room H-3407A, 40 Ruskin Street, Ottawa, ON, Canada K1Y 4W7; Division of Cardiac Surgery, University of Ottawa Heart Institute, 40 Ruskin Street, Ottawa, ON, Canada K1Y 4W7; Division of Cardiology, University of Ottawa Heart Institute, Room H-3407A, 40 Ruskin Street, Ottawa, ON, Canada K1Y 4W7; Division of Cardiology, University of Ottawa Heart Institute, Room H-3407A, 40 Ruskin Street, Ottawa, ON, Canada K1Y 4W7; Department of Cardiovascular Medicine, Mayo Clinic, 200 1st St SW, Rochester, MN 55905, USA; Division of Cardiology, University of Ottawa Heart Institute, Room H-3407A, 40 Ruskin Street, Ottawa, ON, Canada K1Y 4W7; Rambam Health Care Centre, 8 HaAliyah HaShniya Street, Bat Galim, Haifa 3109601, Israel; Division of Cardiology, University of Ottawa Heart Institute, Room H-3407A, 40 Ruskin Street, Ottawa, ON, Canada K1Y 4W7; Division of Cardiology, University of Ottawa Heart Institute, Room H-3407A, 40 Ruskin Street, Ottawa, ON, Canada K1Y 4W7

**Keywords:** Aortic regurgitation, Echocardiography, Mechanism, Aetiology, Aorta, Cardiac surgery

## Abstract

**Aims:**

Aortic regurgitation (AR) arises from leaflet disease and/or dilatation of the functional aortic annulus complex. Understanding the mechanisms of AR informs surgical planning of valve and aorta repair. This study investigates the mechanisms, aetiologies, and outcomes of isolated native severe AR in a consecutive cohort of patients.

**Methods and results:**

Patients with moderate-to-severe (3+)/severe (4+) native valve AR, identified from our institutional echocardiography database (2014–2018), were included. Exclusions were significant concomitant valve disease, endocarditis, or aortic dissection. AR was classified per the El-Khoury system: Type I (normal leaflet motion: Ia–ascending aorta/sinotubular junction dilatation, Ib–aortic root dilation, Ic–aortic annular dilation), Type II (leaflet prolapse), and Type III (leaflet restriction). Valve anatomy and clinical outcomes, including mortality and surgical intervention, were analyzed. Of 282 patients (77.3% male), 58.5% had multiple AR mechanisms. Type II (leaflet prolapse) was most common (48.6%), followed by Type III (36.2%). Bicuspid aortic valve (BAV) represented 35.5% of the population, with leaflet prolapse observed in 72%. Multiple mechanisms were more frequent in BAV (77% vs. 48%, *P* < 0.001). After a median follow-up of 4.7 years (available for 97.5% of patients), 158 (57.5%) underwent an intervention with 48.7% having an aortic valve repair or valve-sparing aortic root replacement.

**Conclusion:**

Although leaflet prolapse (Type II) was the pre-dominant AR mechanism, multiple contributing mechanisms were often present, particularly in BAV patients. Aortic valve repair accounted for nearly half of surgical interventions, underscoring the importance of mechanism identification to optimize repair and avoid valve replacement.

## Graphical Abstract

**Graphical Abstract:**
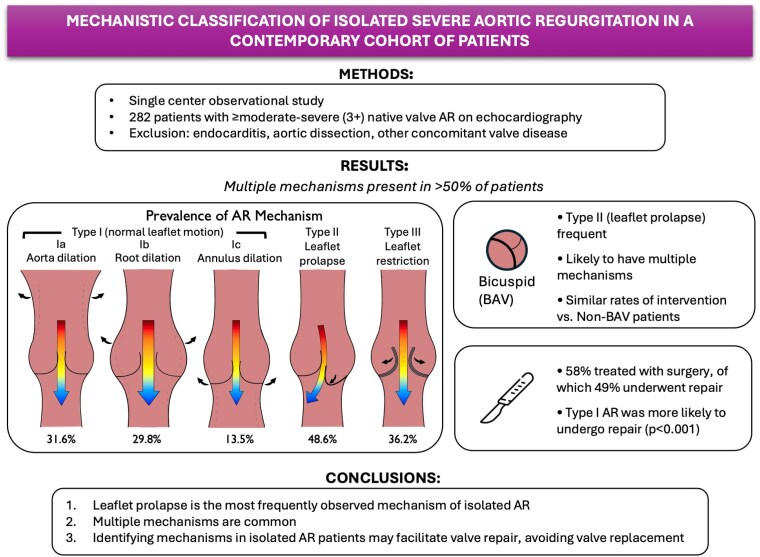

## Introduction

With an estimated prevalence of ∼1–2%, AR is the third-most common valvular heart disease (VHD) following aortic stenosis and mitral regurgitation (MR). The El-Khoury system classifies AR according to leaflet motion into normal aortic valve leaflet motion (Type I, further subdivided into Ia-ascending aorta dilation, Ib-aortic root dilation, and Ic-annular dilation), excessive leaflet motion/prolapse (Type II), and restricted leaflet motion (Type III).^1^ A detailed description of the mechanism of aortic regurgitation (AR) facilitates surgical decision-making. AR mechanisms have been examined in surgical cohorts^2-4^ but rarely in the general population. We aimed to describe the mechanisms of isolated AR in an echocardiography-derived cohort of patients and examine mid-term outcomes associated with each AR type.

## Methods

### Study participants

All patients who underwent an echocardiography at the University of Ottawa Heart Institute between 1 January 2014 and 15 January 2019 with moderate-to-severe (3+) or severe (4+) AR were identified. Patients with other concomitant significant VHD (>mild aortic or mitral stenosis, >moderate MR), and/or prior cardiac/aorta surgery were excluded. AR secondary to infective endocarditis or aortic dissection was also excluded. All echocardiograms and the electronic medical records of included patients were reviewed to identify demographics and patient characteristics, date of last follow-up, survival, dates and type of valve surgery performed.

### Echocardiographic analysis

AV anatomy, including number of cusps, raphe presence and location, and aortic dimensions was identified and measured in accordance with the American Society of Echocardiography guidelines.^5^ Cut-offs for aortic root and ascending aorta enlargement were established using the age, sex, and body surface area reference values from the most recent guidelines.^6^ The aortic annulus was considered enlarged if its diameter exceeded 25 mm.

AR aetiology was assessed based on leaflets’ anatomy, aorta dimensions and the presence of advanced degenerative features or a history of systemic illness. Aortic valve (AV) prolapse/flail leaflet was defined as a malcoaption of the tip of culprit cusp in diastole below the adjacent leaflet with an effective height of ≤ 9 mm.

### Statistical analysis

Comparisons between groups were performed using the Welch’s *t*-test, χ^2^ test The Kruskall-Wallis test followed by Wilcoxon rank-sum tests was used to identify differences in median age based on valve anatomy. A two-sided *P*-value ≤0.05 was used to define statistical significance.

## Results

### Patient cohort and characteristics

**Figure 1:**
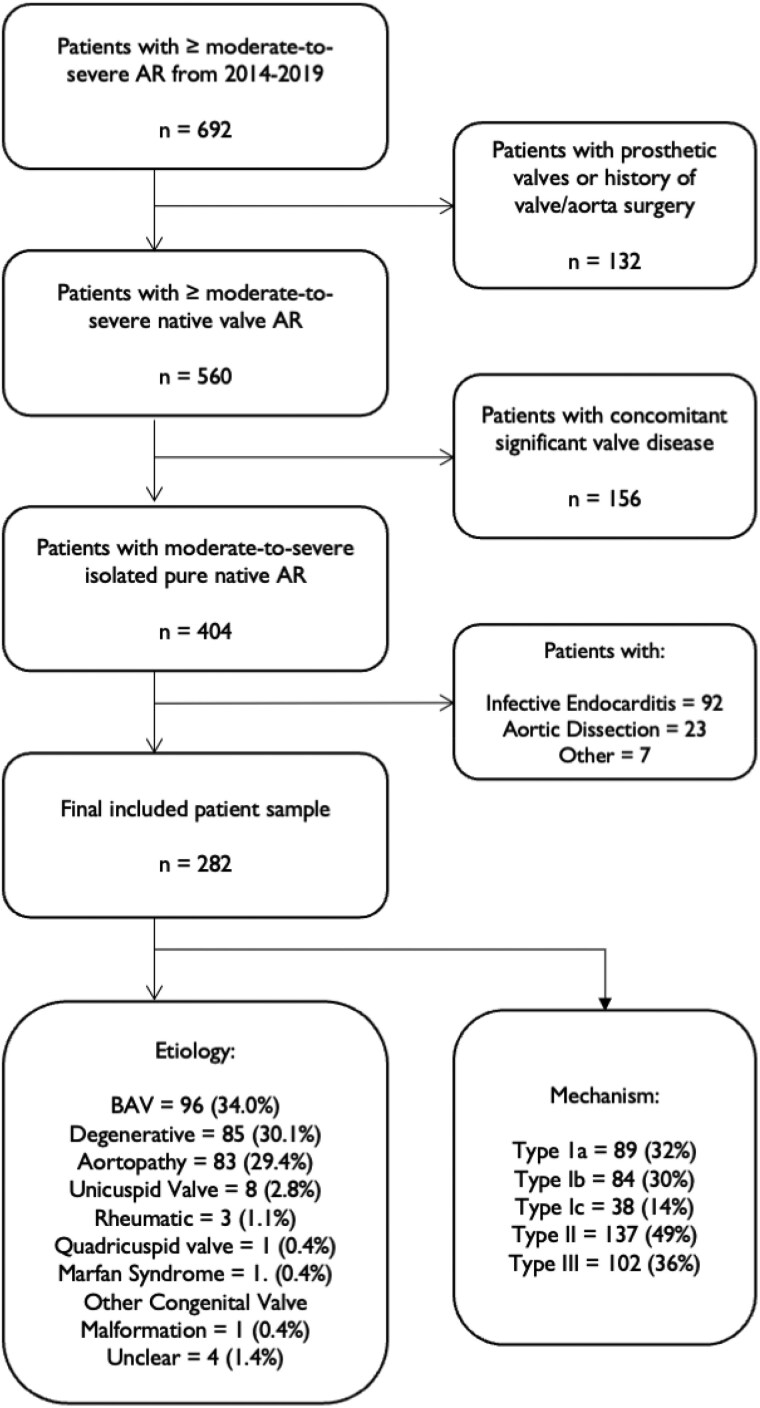
Patients with isolated moderate-to-severe (3+) or severe (4+) aortic regurgitation identified on transthoracic or transoesophageal echocardiography. AR, aortic regurgitation; Ao, aortopathy; Rheum, rheumatic; BAV, Bicuspid aortic valve; CVA, congenital valve abnormality; Deg, degenerative/other; QAV, quadricuspid aortic valve; UAV, unicuspid aortic valve; Unc, unclear.

Of the 86 358 echocardiograms performed at our centre between 1 January 2014 and 15 January 2019, 692 patients presented with moderate-to-severe or severe AR. After the exclusion of prior AV surgery, concomitant VHD and endocarditis or dissection 282 patients were identified (*Figure 1*). The characteristics of the study cohort are described in *Table 1*.

**Table 1:** Demographic and echocardiographic characteristics of patients with aortic insufficiency

Characteristic	Patients (*N* = 282)	BAV (*N* = 96)	Degenerative (*N* = 85)	Aortopathy (*N* = 83)
Age, mean (SD), y	59.6 (17.4)	47.7 (15.6)	68.6 (10.6)	67.6 (13.8)
Male, *N* (%)	218 (77.3)	83 (86.5)	62 (72.9)	61 (73.5)
BMI, median (IQR), kg/m^2^	25.9 (23.4–29.6)	26.0 (5.4)	26.0 (6.3)	26.0 (6.4)
Pulse pressure, MMHG (IQR)	70 (59–87)	68 (23)	75 (29)	77 (32)
Severity of AR, *N* (%)				
3+ AR	176 (62.4)	57 (59.4)	59 (69.4)	49 (59.0)
4+ AR	106 (37.6)	39 (40.6)	26 (30.6)	34 (41.0)
Valve anatomy, *N* (%)				
Tricuspid	160 (56.7)	0 (0)	77 (90.6)	77 (92.8)
Bicuspid	100 (35.5)	96 (100)	1 (1.2)	2 (2.4)
Right/left coronary cusp fusion	81	73	1	2
Right/non-coronary cusp fusion	13	13	0	0
Left/non-coronary cusp fusion	2	2	0	0
Unclear	4			
Unicuspid	8 (2.8)	0 (0)	0 (0)	0 (0)
Quadricuspid	2 (0.7)	0 (0)	0 (0)	1 (1.2)
Unclear	12 (4.3)	0 (0)	7 (8.2%)	3 (3.6)
LV function, *N* (%)				
Normal LV function	191 (67.7)	74 (77.1)	56 (65.9)	48 (57.9)
Borderline LV dysfunction	25 (8.9)	8 (8.3)	6 (7.1)	8 (9.6)
Mild LV dysfunction	38 (13.5)	10 (10.4)	11 (12.9)	15 (18.1)
Moderate LV dysfunction	16 (5.7)	2 (2.1)	6 (7.1)	8 (9.6)
Severe LV dysfunction	10 (3.6)	2 (2.1)	5 (5.9)	3 (3.6)
Unclear	2 (0.7)	0 (0)	1 (1.1)	1 (1.2)

Abbreviations: AR, aortic regurgitation; BMI, body-mass index; IQR, interquartile range; LV, left ventricle.

### Aetiology of AR

The most common aetiologies of AR were bicuspid aortic valve (BAV) in 96 patients (34.0%), degenerative in 85 patients (30.1%), and aortopathy in 83 patients (29.4%). Other aetiologies included unicuspid valves in patients 8 (2.8%), rheumatic AV disease in 3 (1.1%) patients, a quadricuspid valve in 1 (0.4%) patient, Marfan syndrome in 1 (0.4%) patient and malformation (0.4%) in one patient. In 4 (1.4%) patients, the aetiology was unclear.

### Mechanisms of AR

A single AR mechanism was noted in 115 patients (40.8%), while 165 patients (58.5%) had multiple mechanisms. In 2 (0.7%) patients, the mechanism could not be determined (*Table 2*). Type II (leaflet prolapse) was the most frequently encountered mechanism seen in 137 (48.6%) of patients, followed by Type III (leaflet restriction) in 102 (36.2%) of patients. Type Ia (dilated ascending aorta) AR was seen in 89 (31.6%) patients, Type Ib (dilated aortic root) in 84 (29.8%) patients, and Type Ic (dilated aortic annulus) in 38 (13.5%) patients.

**Table 2:** Mechanism and aetiology of aortic regurgitation

Mechanism^a^	*N*	Age	BAV	Rheum	Ao	Deg	Unsure	UAV	QAV	Marfan	CVA
IA	30	66.2	0	0	28	2	0	0	0	0	0
IB	23	68.8	2	0	21	0	0	0	0	0	0
IC	1	26	0	0	1	0	0	0	0	0	0
III	39	66.2	8	1	1	26	3	0	0	0	0
IA + II	26	61.3	15	0	3	7	0	1	0	0	0
IA + III	32	63.5	7	0	6	15	0	3	1	0	0
IB + II	45	51.8	22	0	6	16	0	0	0	1	0
IB + III	13	65.2	5	2	2	4	0	0	0	0	0
IC + II	31	42.8	20	0	0	9	0	1	0	0	1
IC + III	5	54.8	2	0	0	2	0	1	0	0	0
II + III	8	70.6	3	0	0	5	0	0	0	0	0
IA + II + III	1	69	0	0	0	1	0	0	0	0	0
IB + II + III	3	44	1	0	0	1	0	1	0	0	0
IC + II + III	1	26	0	0	0	0	0	1	0	0	0
Unclear	2	53.5	1	0	0	0	1	0	0	0	0

^a^As per El-Khoury classification: Type I, normal leaflet motion; Type Ia, dilated ascending aorta and sinotubular junction; Type Ib, dilated aortic root; Type Ic, dilated aortic annulus; Type II, leaflet prolapse; Type III, leaflet restriction.

Among the 165 patients with multiple AR mechanisms, the most common combination was Type Ib and II, seen in 45 (27.3%) patients, followed by Type Ia and III in 32 (19.4%) patients. Five (3.0%) patients had three distinct mechanisms of AR. Among the 115 patients with single mechanisms of AR, Type III was the most common in 39 patients (33.9%), followed by Type Ia in 30 (26.1%) of patients, Type Ib in 23 (19.7%), and Type II in 22 (18.8%). One (0.1%) patient had isolated Type Ic AR. One hundred (35.5%) of patients had a BAV. The presence of Type II AR (72% vs. 28%, *P* < 0.001), as well as AR due to multiple mechanisms (77% vs. 48%, *P* < 0.001), was more common in patients with than without BAV.

### Severity of AR

Patients with Type II AR were more likely to have severe (4+) AR than those without (50.3% vs. 25.6%, *P* < 0.001), whereas patients with leaflet restriction were less likely to have severe (4+) AR than those without (18.6% vs. 48.3%, *P* < 0.01). No significant differences in severity of AR were found between aetiology of AR, number of cusps, or number of mechanisms of AR.

### Interventions and mortality

During a median follow-up of 4.7 years, IQR (3.0–6.6) [available in 275 (97.5%) patients], 19 patients (6.9%) died, and 158 patients (57.5%) underwent AV surgery, which comprised 77 (48.7%) AV repair or valve-sparing aortic root replacement, and 80 (50.6%) patients AV replacement [mostly (82.5%) bioprosthetic valve]. One patient with severe AR and mild stenosis underwent a transcatheter aortic valve implantation. Rates of any intervention were similar between patients with and without BAV (58.0% vs. 57.1%, *P* = 0.89) that persisted after adjustment for age, sex and AR severity.

The rate of valve repair/valve-sparing aortic root repair varied with AR type and was higher in Type I AR compared with other types (56% vs. 21%, *P* < 0.001). For patients with Type II AR, the rate was similar to those other types (54% vs. 41%, *P* = 0.1), while the rate was lower in Type III compared with other types (19% vs. 60%, *P* < 0.001). The rate of valve repair was similar among patients with and without BAV (51.7 vs. 48.3%, *P* = 0.6).

## Discussion

This study reports mechanisms of isolated AR using the El-Khoury classification in a non-surgical cohort. Surgical cohorts of patients with isolated AR have previously described the mechanism of AR but are highly selected.^2-4^ Compared with prior surgical reports, our patient population was older, which was likely driven by the inclusion of patients who were either non-surgical candidates or in whom the risk-benefit ratio favoured a conservative approach. Presence of a BAV was common (34–44%) in our and prior studies, reflecting the relatively high frequency of this abnormality as an aetiology for AR. As in our study, prolapse was found to be the most common AR mechanism in surgical studies that specifically reported on this finding. The presence of multiple mechanisms was somewhat higher in our study. This difference may be driven by our older population with an associated higher rate of degenerative leaflet disease complicating root or ascending aorta dilatation. Our study also found that the most common mechanistic combination type was root dilatation and prolapse.

### Surgical intervention and outcomes

Over a 4.7-year period, over half of the included patients underwent a surgical intervention, of which close to half were an AV repair/annuloplasty. This high rate of valve-sparing repair reflects growing recognition of the feasibility, and favourable outcomes in selected patients, as well as our local expertise. Despite the increasing volume of AV repair reported in the literature, the general current rates are not well described and are centre and operator dependent.

### Limitations

Our study has several limitations, including (i) being a single-centre retrospective design, (ii) enrolment based on index echocardiogram which included both transthoracic echocardiogram or transesophageal echocardiogram, (iii) exclusion of patients with AV endocarditis (which explains why we did not identify patients with Type Id AR), (iv) AV repair is only performed by highly experienced surgeons at our site and our results may not be applicable to all centres or regions, and (v) patients deemed not to be surgical patients were not identified in the data review process. In addition to echocardiography, cross-sectional imaging such as CT and MRI are frequently used in further determining the presence of aortic root and ascending aorta dilatation. The potentially complementary role of these advanced cardiac imaging techniques for the classification of the mechanism of severity of AR was also not assessed in this study.

## Conclusion

We report AR aetiologies and mechanisms in a consecutive cohort of patients with isolated AR identified through echocardiography at a quaternary cardiac care centre. Leaflet prolapse was the most common AR mechanism, followed by leaflet restriction. Most patients had multiple mechanisms, especially those with BAV, who represented one-third of the population. The most common combinations were aortic root dilatation and leaflet prolapse, followed by leaflet restriction and ascending aorta dilatation. AV repair was performed in about half of all patients.

## Data Availability

The data underlying this article will be shared on request to the corresponding author.

## Lead author biography

Dr. Beauchesne is a full-time staff cardiologist at the Ottawa Heart Institute and is a Professor of Medicine at the University of Ottawa. He completed his medical studies at McGill University and his postgraduate training at Dalhousie University in Halifax, Foothills Hospital in Calgary, the Ottawa Heart Institute and at the Mayo Clinic in Minnesota. Dr. Beauchesne is a Fellow of the Royal College of Physicians and Surgeons of Canada and the American College of Cardiology. He is a member of the Canadian Cardiovascular Society, the Canadian Society of Echocardiography, the American Society of Echocardiography, the Canadian Adult Congenital Health Network and the International Society of Adult Congenital Heart Disease. His research interests focus on adult congenital heart disease, valvular heart, aortopathy and echocardiography.
